# Evaluation of a Cognitive Aid Application to Improve Non-Technical Skills in Simulated Cardiopulmonary Resuscitation (CPR): A Randomised Controlled Trial

**DOI:** 10.3390/clinpract16040069

**Published:** 2026-03-30

**Authors:** Carlos Ramon Hölzing, Tristan Ernst, Thomas Wurmb, Tobias Grundgeiger, Patrick Meybohm, Oliver Happel

**Affiliations:** 1 Department of Anaesthesiology, Intensive Care, Emergency and Pain Medicine, University Hospital Würzburg, Oberdürrbacher Str. 6, 97080 Würzburg, Germanyhappel_o@ukw.de (O.H.); 2 Psychological Ergonomics, Julius-Maximilians-University of Würzburg, 97070 Würzburg, Germany

**Keywords:** resuscitation, emergency medicine, patient simulation, mobile applications, clinical competence, leadership

## Abstract

Background/Objectives: The success of cardiopulmonary resuscitation relies on both technical and non-technical skills. Cognitive aids, such as checklists, have been shown to enhance technical performance in emergencies. The aim of this study was to evaluate the capabilities of a cognitive aid app (CA-App) in improving non-technical skills. Methods: In this single-centre randomised controlled trial, 62 teams, each consisting of an experienced physician and a specialised nurse, were randomised either to CA-App or control (No-App) groups performing cardiopulmonary resuscitation. The study was registered with the German Clinical Trials Register (DRKS) on 4 November 2025 (DRKS00038336). The primary outcome was the team leader’s performance in non-technical skills, assessed via the validated Team Emergency Assessment Measure (TEAM™) questionnaire by two raters. Secondary analyses examined TEAM™ subdomains (leadership, teamwork, task management) and the correlation between app usage duration and performance. Results: 62 out of 67 teams were finally randomised, with 31 teams in each group. The CA-App group demonstrated a marginally elevated median TEAM™ total score (83.33%) in comparison to the control group (79.33%), although this difference was not statistically significant (*p* = 0.190). The leadership subgroup score was significantly higher in the app group (*p* = 0.006). There was no significant correlation between the time spent using the app and improved team performance (r = 0.260, *p* = 0.166). Conclusions: The CA-App demonstrated potential for improving leadership skills, a critical component of non-technical skills in emergency scenarios. These findings highlight the potential capability of cognitive aids to improve non-technical skills and the need for further research to explore their optimal design and integration into clinical practice to enhance team performance and patient safety.

## 1. Introduction

The efficacy of cardiopulmonary resuscitation (CPR) is contingent upon the seamless integration of technical and non-technical skills. Technical skills are pivotal in ensuring the provision of high-quality chest compressions and effective ventilation, thereby maintaining circulation and oxygenation during cardiac arrest. Higher-quality chest compressions have been shown to improve survival rates and neurological outcomes in patients experiencing cardiac arrest [1,2]. These technical skills deteriorate rapidly without consistent training [3]. This finding indicates that ongoing training is a crucial element in preserving technical proficiency [4]. In addition to technical skills, medical care requires non-technical skills (NTS) such as communication, teamwork, decision-making and situation awareness [5,6]. NTSs also have a positive impact on technical skills and are particularly important in CPR [7,8]. Inadequacy in NTS has been associated with adverse patient outcomes, underscoring the need for effective training and support tools [9,10]. As asserted by Peran et al. and Hunziker et al., greater situational awareness and more effective task management facilitate improved adaptation to dynamic clinical conditions, optimise decision-making, and support adherence to established guidelines [11,12].

The integration of cognitive aids (CA), such as checklists and flowcharts, may support both the technical and non-technical dimensions of performance in resuscitation. CAs represent sequenced, evidence-based recommendations designed to reduce cognitive load and support adherence to guidelines, potentially limiting error seven during high-pressure events [13,14]. The implementation of CAs during CPR was associated with improved compliance with Advanced Cardiac Life Support (ACLS), reduced failure in executing critical steps, and overall improvements in team performance [15]. Moreover, studies have demonstrated that the use of cognitive aids results in improved adherence to best practices in the management of deteriorating surgical patients, leading to a significant reduction in omitted critical management steps [16]. Despite these advances, the specific impact of cognitive aids on the team leader’s non-technical skills during resuscitation remains insufficiently understood. Previous studies have primarily examined technical performance or overall team behaviour without isolating the leader’s contribution.

This research investigates how well a CA application can improve a team leader’s non-technical skills in simulated emergency medical scenarios. We hypothesised that the intervention group using the CA-App would show significant improvements in NTS as measured using the Team™ questionnaire compared with a control group without the app. Secondary aims were to assess app usage time and subsequent impacts on adherence to guidelines and workflow efficiency. Therefore, this study will investigate the potential of mobile cognitive aids to improve NTS to fill a critical gap in resuscitation science and inform the development of innovative strategies to improve patient outcomes in emergency care.

## 2. Materials and Methods

This section outlines the design, setting, participants, interventions, outcomes, sample size calculation, randomisation, and statistical methods used in this study. The study was conducted in accordance with the CONSORT guidelines [17], (Supplement File S1).

### 2.1. Trial Design

This study was a single-centre, randomised controlled trial (RCT) employing a parallel group design to evaluate the effectiveness of a cognitive aid application (CA-App Version 1.0) in improving non-technical skills (NTS) during simulated emergency medical scenarios. The data analysed here are identical to those reported by Grundgeiger et al. where a separate primary endpoint, a 12-variable CPR performance score, was examined to evaluate the app’s impact on guideline adherence and quality of CPR [18]. The study was registered with the German Clinical Trials Register (DRKS) on 4 November 2025 (DRKS00038336). Prospective registration was not required, as this simulation-based study involved no patients, medicinal products, or medical devices and therefore did not meet the definition of a clinical trial under the German GCP regulation applicable at the time.

### 2.2. Setting

The study was conducted between April and December 2017 in the simulation centre of the Department of Anaesthesiology, University Hospital Würzburg. The simulations were performed using the Resusci Anne^®^ resuscitation simulator (Laerdal, Stavanger, Norway) to replicate a realistic cardiovascular arrest scenario based on the European Resuscitation Council (ERC) algorithm. Each team comprised one physician and one nurse, supported by two certified paramedics who were trained in basic life support (BLS). The paramedics were the same individuals across all simulations and were instructed to act in a consistent, scripted manner, limiting their interactions to BLS and mechanical support to ensure standardisation. The paramedics performed predefined supportive tasks only, including chest compressions, preparation of medication syringes, and mechanical assistance as instructed by the team leader. They did not assume leadership roles or make independent clinical decisions. The paramedics were not instructed to interact with the cognitive aid application and did not receive visual access to the tablet display. Their behaviour followed a fixed script across all simulations to minimise variability. Each simulation consisted of seven rhythm analyses with one shockable rhythm (ventricular fibrillation) and concluded with return of spontaneous circulation (ROSC). This study was conducted in accordance with the Declaration of Helsinki and was approved by the Ethics Committee of the University of Würzburg on 28 March 2017 (approval no. 40/17-ge).

### 2.3. Participants

Participants were recruited via internal communications at the University Hospital Würzburg, both Departments of Anaesthesiology and Internal Medicine. Each team consisted of a physician and a nurse. The eligibility criteria for physicians included formal qualifications in emergency medicine in addition to a minimum of two years of professional experience. Registered nurses were required to be currently working in an area of acute medicine, such as anaesthesia, emergency, or intensive care nursing, to qualify for participation. All participants provided written informed consent prior to inclusion in the study, including consent for the use and publication of anonymised data.

### 2.4. Intervention

The intervention group was provided with a tablet device equipped with the CA-App, which was developed and evaluated by Grundgeiger et al. [14]. The CA-App was installed on an Android-based tablet device (Sony Xperia Z2 (Sony Corporation, Tokyo, Japan), 10.1-inch display). The tablet was handed to the designated team leader at the beginning of the scenario. The team leader was instructed to hold the device during resuscitation. The screen was primarily visible to the team leader and was not intended as a shared team display. The application did not progress automatically through the resuscitation algorithm. All relevant events (e.g., rhythm check, defibrillation, medication administration, airway intervention, ROSC) required active manual input by the team leader.

Interaction with the application occurred directly via touch input during active CPR. No hands-free or voice-controlled operation was implemented. Upon initiation of a resuscitation scenario, the team leader activated the protocol and confirmed arrival at the patient. The main interface then displayed key time-dependent and algorithm-relevant information. The screen continuously showed the elapsed time since patient arrival, the time since the last rhythm check, and the time since the last adrenaline administration.

During each CPR cycle, the app displayed algorithm-aligned visual guidance based on the European Resuscitation Council (ERC) guidelines. A rhythm check timer counted down the recommended interval and changed colour at predefined thresholds (e.g., 1:40 and 2:00 min), accompanied by vibration alerts. Documenting a rhythm check reset the timer. After the third and fifth defibrillation, a syringe icon appeared to indicate consideration of amiodarone administration. Colour-coded indicators signalled when repeat adrenaline dosing should be considered.

**Figure 1:**
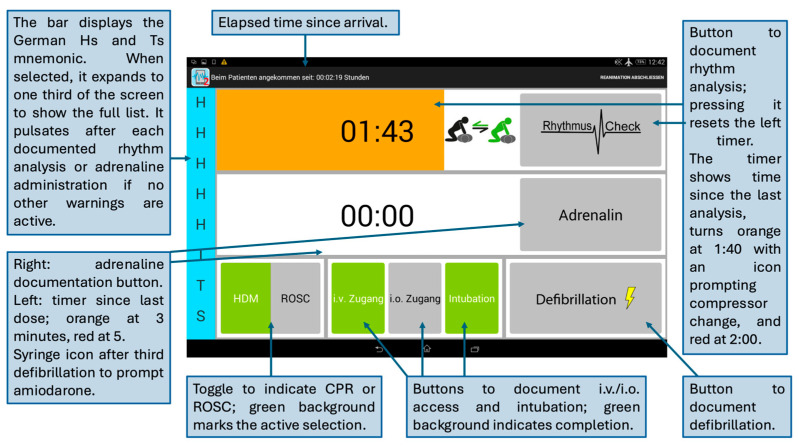
Screenshot of the cognitive aid application during simulated cardiopulmonary resuscitation.

The interface included dedicated icons for documentation of chest compressions, defibrillation, airway interventions (e.g., intubation), medication administration, and return of spontaneous circulation (ROSC). All entries were automatically timestamped. An additional icon reminded the team to rotate the provider performing chest compressions at appropriate intervals. A collapsible display of the German version of the “Hs and Ts” mnemonic supported structured assessment of reversible causes following rhythm analysis. The application did not perform automated drug dosage calculations [14,18,19]. A screenshot of the application interface is presented in Figure 1.

Prior to the simulation, participants in this group underwent a brief orientation session, spanning three minutes, with the objective of familiarising with the app’s features. They were instructed to utilise the app throughout the scenario and were encouraged to interact with it as frequently as necessary. The control group, meanwhile, performed the CPR task without any digital assistance. It is noteworthy that both groups adhered to the same simulation protocols, ensuring consistency in the clinical scenario across participants.

### 2.5. Outcomes

The primary outcome was improvement in the team leader’s non-technical skills, assessed using the validated Team™ questionnaire [20]. All TEAM™ ratings were performed using video recordings of the simulation scenarios. The complete resuscitation scenarios were recorded and later independently reviewed by two raters. Assessments were conducted after completion of data collection and were not performed live during the simulations. Blinding of raters to group allocation was not feasible, as use of the tablet device was clearly visible in the video recordings. Raters were aware that one group used the cognitive aid application and one did not.

The TEAM™ instrument comprises 11 behavioural items rated on a 0–4 Likert scale (0 = never 4 = always) and one global rating scored from 1 to 10. An overview of all items, their subdomains, and rating scales is provided in Table 1.

In accordance with the TEAM™ framework, items were analysed both as a total score (sum of items 1–12, maximum 54 points, converted to percentage values for reporting) and within three predefined subdomains: leadership (items 1–2), teamwork (items 3–9), and task management (items 10–11). For the subdomains, a conversion to percentage of the maximum possible points was performed as well. This tool assesses key dimensions of NTS, including leadership, communication, and decision-making. Secondary outcomes included total app usage time in the intervention group and the potential relationship between app usage and NTS scores. Performance ratings were conducted by two independent assessors with advanced training in human factors and resuscitation education. Prior to data collection, interrater calibration sessions were held to ensure consistent application of the TEAM™ rating criteria. Although no formal structured training programme was conducted prior to rating.

### 2.6. Sample Size and Randomisation

Preliminary studies indicate a substantial impact of the application (d = 0.8) [14,18]. A power analysis (1 − β = 0.8, α = 0.05) determined that a total of 66 participants were needed (33 in each group) using a Mann–Whitney U test. Randomisation was performed using a computer-generated random sequence. Teams were allocated either to the intervention or control group in a 1:1 ratio. Due to the nature of the intervention, blinding of participants was not feasible.

### 2.7. Statistical Methods

Statistical analyses were performed using R software (version 4.2.1). Given the non-normal distribution of the data, confirmed by the Shapiro–Wilk test, non-parametric methods were used. The Mann–Whitney U test was used to compare primary and secondary outcomes between groups, with a significance threshold of α = 0.05. The Bonferroni correction was used for a total of four comparisons resulting in a corrected significance threshold of α = 0.0125. Glass’s rank-biserial correlation (r_g_) was chosen as the effect size measure for the Mann–Whitney U test. Spearman’s rank correlation coefficient was used to explore relationships between NTS scores and variables such as professional experience, technical skills, cognitive load and time spent using the application.

Two independent raters assessed each participant’s performance using the Team™ questionnaire. Interrater reliability was evaluated with the intraclass correlation coefficient (ICC) using a two-way random-effects model. The ICC for single measures was 0.660 (95% CI [0.507, 0.773]) and for average measures 0.795 (95% CI [0.673, 0.882]). The reliability was statistically significant (F(71,71) = 4.882, *p* < 0.001), indicating good agreement between raters. Cronbach’s Alpha was 0.795, reflecting good internal consistency.

## 3. Results

**Figure 2:**
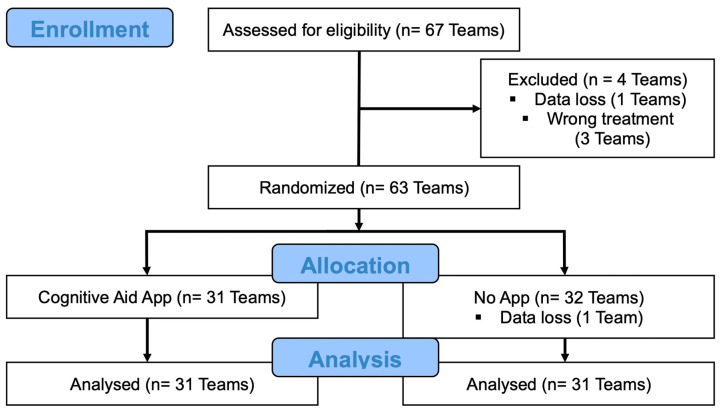
CONSORT flow diagram.

A total of 67 teams, each consisting of one physician and one nurse, were recruited. Four teams were excluded before analysis due to technical problems during simulation or randomisation. In one case, a malfunction in the simulator control led to a deviation from the intended resuscitation sequence (“wrong treatment”), and three cases were affected by incomplete or non-assignable questionnaire data (“data loss”). Consequently, 63 teams were successfully randomised: 31 teams were assigned to the intervention group (CA-App) and 32 teams to the control group (No-App). One team in the control group was subsequently excluded due to data loss, resulting in 31 teams per group for final analysis (see Figure 2).

### 3.1. Participant Characteristics

As displayed in Table 2, the demographic and professional characteristics of subjects within each group were equivalent. One participant who was part of the No-App group did not complete the demographic survey and was excluded from that specific analysis. There were no significant differences between the groups with regard to gender distribution, age, or professional experience for either physicians or nurses.

### 3.2. TEAM™ Questionnaire Results

**Figure 3:**
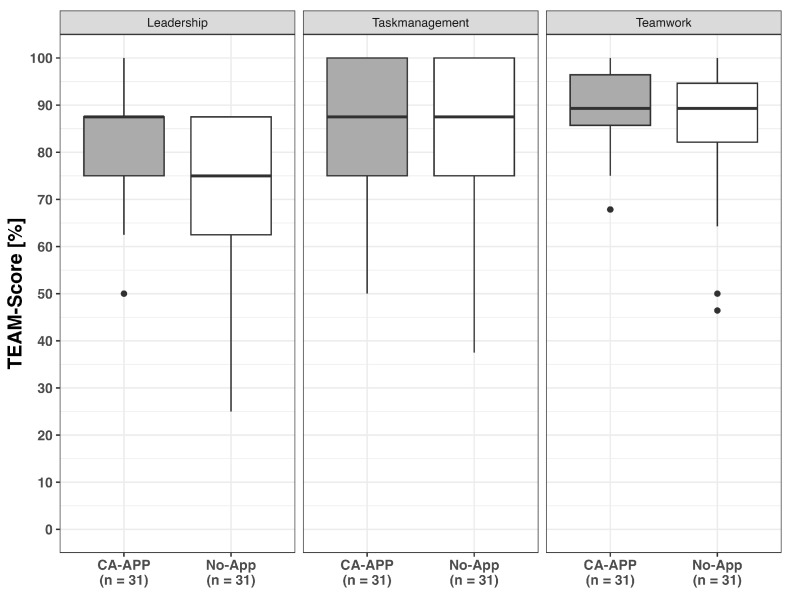
TEAM™ subgroup scores (leadership, teamwork, task management) for CA-App and No-App groups.

The mean TEAM™ total score for the CA-App group (*n* = 31) (Mdn = 83.33%, IQR = 18.52) was marginally higher than for the No-App group (*n =* 31) (Mdn = 79.63%, IQR = 13.89), yet this difference was not statistically significant (U = 574.0; *p* = 0.190; 95% CI [−1.852, 9.259]; r_g_ = 0.195). As a secondary outcome measure, the 12 TEAM™ items were analysed by categorising them into three subgroups (leadership, teamwork, and task management) to test the hypothesis that the CA-App would affect scores within the TEAM™ subgroups. The leadership subgroup exhibited a significantly higher score with the CA-App (*n =* 31) (Mdn = 87.5%, IQR = 12.5) in comparison to the No-App (*n =* 31) (Mdn = 75%, IQR = 25.0) (U = 670.5; *p* = 0.006; 95% CI [0.0, 25.0]; r_g_ = 0.395). Statistical significance was preserved after correction for multiple comparisons using the Bonferroni method (adjusted significance threshold α = 0.05/4 = 0.0125). There was no significant difference between the task management subgroup in the CA-App group (*n =* 31) (Mdn = 87.5%, IQR = 25.0) and the No-App group (*n =* 31) (Mdn = 87.5%, IQR = 25.0) (U = 541.5; *p* = 0.376; 95% CI [0.0, 12.5]; r_g_ = 0.127). The analysis further revealed no significant variation in teamwork between the CA-App Group (*n =* 31) (Mdn = 89.29%, IQR = 10.71) and the No-App Group (*n =* 31) (Mdn = 89.29%, IQR = 12.5) (U = 527.5; *p* = 0.508; 95% CI [−3.571, 7.143]; r_g_ = 0.098) (see Figure 3).

**Figure 4:**
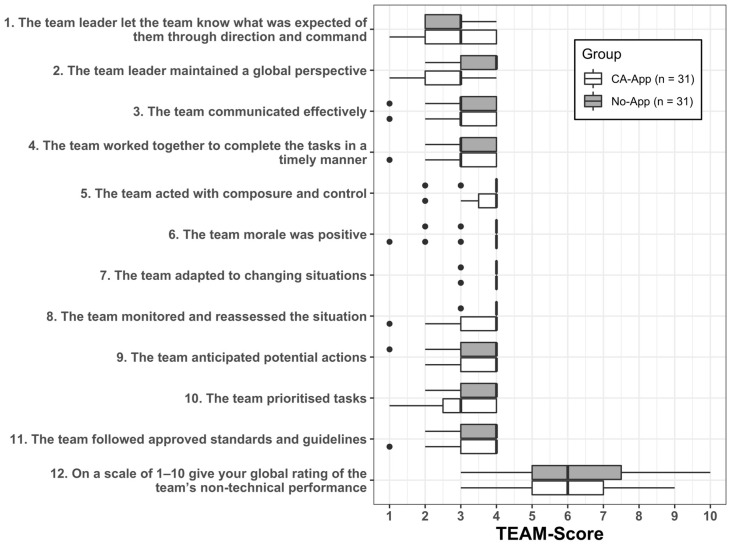
TEAM™ item scores comparing CA-App and No-App groups.

A further specific evaluation of the questionnaire items showed different medians between the CA-App and the No-App group in item 2 (“The team leader maintained the global perspective”) and item 10 (“The team prioritised tasks”). Figure 4 provides a graphical representation of these findings.

### 3.3. TEAM™ Correlation and App Usage

A correlation analysis was conducted on the TEAM™ total score and the team leader’s professional experience, yielding an inverse relationship with a correlation coefficient (r = −0.472, *p* < 0.001, 95% CI [−0.770, −0.246]), suggesting a medium effect size according to Cohen’s criteria. Within the CA-App group, a correlation analysis was performed between app uses per minute and total TEAM™ score (M = 1.925/min, IQR = 0.595/min, Range = 1.998/min, SD = 0.443/min), yielding a correlation coefficient of r = 0.260, *p* = 0.166, 95% CI [−0.111, 0.567], meaning that there was no direct relationship between the amount of CA-App usage and improvement in team performance scores.

## 4. Discussion

The present study constitutes a valuable addition to the field by offering a cognitive aid application that can be used to enhance the performance of residents in non-technical skills within simulated emergency medical scenarios.

The primary objective of this study was to assess the effectiveness of the CA-App in enhancing team leaders’ NTS in emergency scenarios. The study did not demonstrate a statistically significant improvement in the primary endpoint, namely the overall TEAM™ total score (*p* = 0.190). Thus, with respect to its primary outcome, this trial must be considered negative. Interrater reliability for single measures was moderate (ICC = 0.660), indicating acceptable but not excellent agreement. Although reliability for averaged ratings was higher, variability in individual assessments may have reduced statistical sensitivity for detecting true differences between groups. Behavioural rating instruments such as TEAM™ require subjective judgement, particularly when evaluating leadership and communication behaviours, and some interpretative variation cannot be fully eliminated despite structured rater training. While the intervention group showed a numerically higher median TEAM™ score, this difference was not statistically significant. This result is inconsistent with the findings of Marshall et al., who reported significant enhancements in non-technical skills when using a cognitive aid application in a less familiar and higher-complexity scenario involving a “cannot intubate, cannot oxygenate” crisis. The observed discrepancy between the two studies could be attributed to differences in the scenarios studied. CPR is a frequently trained and more familiar emergency for healthcare providers compared to difficult airway management, potentially diminishing the impact of cognitive aids in this context. In contrast, our findings partly align with those of De Bie Dekker et al., who observed improvements in specific teamwork behaviours and reductions in omitted critical steps through checklist-supported acute care simulations [21].

Similarly, Donzé et al. demonstrated that digital cognitive aids can enhance targeted leadership and situational awareness domains, reinforcing the leader-focused improvement observed in our study [22,23]. These findings underscore the potential of cognitive aids to enhance specific components of NTS. Despite the negative primary outcome, our study demonstrated a significant improvement in the predefined leadership subdomain. This secondary finding suggests a domain-specific effect rather than a global improvement in non-technical skills. The substantial improvement in leadership scores is particularly salient, as effective medical leadership is widely acknowledged as a pivotal factor in enhancing healthcare delivery quality [24]. The app’s simple and intuitive structure, which visually organises CPR procedures, may have facilitated team leaders’ ability to maintain situational awareness and prioritise tasks effectively. Additionally, the built-in timers in the app likely helped users manage cognitive resources more efficiently, supporting better task execution under pressure. Similar benefits of structured cognitive aids have been observed in previous studies, particularly in scenarios in-volving high stakes and time-critical decision-making [14,25,26].

The results have indicated an absence of a significant correlation between the du-ration of app usage and TEAM™ scores, which suggests that extensive app usage does not necessarily lead to enhanced team performance. This finding emphasises the need to strategically integrate cognitive tools into practice, focusing on their efficient and targeted use rather than prolonged dependence. Clinicians should be trained to use cognitive aids as a complement to their expertise rather than as a substitute.

An intriguing finding was the inverse relationship between TEAM™ scores and clinical experience among team leaders (r = −0.472, *p* < 0.001). This suggests that more experienced clinicians achieved lower observable non-technical skill ratings in this simulated setting. Several explanations are conceivable. First, experienced clinicians may rely less on external guidance and structured prompts, including cognitive aids, due to greater internalised procedural knowledge. Second, they may delegate tasks more efficiently, which could reduce overt leadership behaviours captured by the TEAM™ instrument. Third, familiar scenarios such as CPR may be perceived as routine by experienced providers, potentially resulting in lower visible engagement or verbalisation of decision processes. Alternative explanations must also be considered. A ceiling effect among less experienced clinicians, who may have been more motivated to perform visibly well in a simulated and observed setting, cannot be excluded. In addition, more experienced clinicians may demonstrate subtle leadership behaviours that are less explicitly verbalised and therefore less detectable by behavioural rating tools. Resistance toward structured cognitive aids or lower affinity for digital tools in more senior participants may also have influenced interaction patterns and observable behaviour. Generational differences in familiarity with mobile applications could have affected how naturally the intervention was integrated into team communication. These interpretations remain speculative and warrant further investigation, particularly in real-world clinical settings.

Although the study was conducted in 2017, the relevance of its findings remains high, as both the structure of CPR protocols and the core principles of non-technical skills have not fundamentally changed in recent years. Moreover, the growing integration of digital cognitive aids into clinical practice underscores the continued applicability of our results.

### 4.1. Implications and Future Directions

The findings of this study suggest that cognitive aids such as the CA-App have the potential to enhance specific components of NTS, particularly leadership, in emergency scenarios. Combined with our previous findings on improving technical skills, these data support the integration of cognitive aids as multifaceted educational tools that simultaneously address procedural accuracy and leadership skills. However, their integration into clinical practice must be approached strategically. Training programmes should focus not only on app functionality but also on its optimal use in dynamic, real-world settings.

Future studies should confirm these results in multi-centre trials across different clinical settings and participant populations. Further research into the adaptation of cognitive aids for users of different levels of experience may lead to better uptake and use. Design changes, including the development of multilingual interfaces and integration with electronic medical records, may also serve to further facilitate their use. The CA-App evaluated in this study was developed for use in real in-hospital resuscitations. Beyond providing structured timing support, the application enables automated, timestamped documentation of key resuscitation events. This facilitates accurate documentation and structured transfer of data into institutional and national in-hospital resuscitation registries.

Longterm impact of the usage of cognitive aids on clinical outcomes, such as patient safety and quality of care, should be examined, as this will be important to the integration of cognitive aids into routine training and practice in emergency medicine.

### 4.2. Limitations

Despite the demonstration of certain beneficial outcomes resulting from such applications, several limitations must be considered when interpreting results. Primarily, the generalisability of the results is inherently limited by the simulated environment. While simulations are controlled and reproducible, they cannot replicate the high-stakes complexity and variability of real-life emergencies [27]. In particular, the absence of real patient harm, emotional stress related to family presence, unexpected equipment failures, or competing clinical demands may have reduced situational pressure compared with actual cardiac arrest scenarios. Furthermore, the nature of the intervention prevents any blinding of participants, thereby presenting a threat of performance bias. Participants may have modified their behaviour due to awareness of being observed (Hawthorne effect), which could have influenced non-technical skill ratings. Because participants were aware of group allocation, behaviour may have been consciously or unconsciously altered, particularly in the intervention group, where the use of a tablet device was visible. This awareness may have increased adherence to structured communication or guideline-based actions independent of the app’s intrinsic effect. The single-centre design of the study limits its generalisability to other healthcare institutions or cultural contexts.

However, when taking into account the study’s limitations, the fundamental results can still provide a valuable basis for further investigations and may offer preliminary guidance for the use of cognitive aids in enhancing non-technical skills in clinical practice.

## 5. Conclusions

The objective of this research was to examine the effectiveness of the cognitive aid application in enhancing NTS in simulated emergency medical scenarios. Although the overall improvement in NTS between the intervention group and the control group was not statistically significant, a marked improvement in the leadership subgroup scores was observed in the CA-App group. These findings underscore the utility of cognitive aids in supporting leadership, a critical component of team performance in high-stakes clinical events.

The study emphasises the necessity for further research to investigate the optimal design and integration of cognitive aids into clinical practice to enhance NTS and team performance.

## Figures and Tables

**Table 1:** Overview Team™ questionnaire item with subdomains and scales.

Team™ Questionnaire Item	Subdomain	Rating
The team leader let the team know what was expected of them through direction and command.	Leadership	0–4
2. The team leader maintained a global perspective.	Leadership	0–4
3. The team communicated effectively.	Teamwork	0–4
4. The team worked together to complete tasks in a timely manner.	Teamwork	0–4
5. The team acted with composure and control.	Teamwork	0–4
6. The team morale was positive.	Teamwork	0–4
7. The team adapted to changing situations.	Teamwork	0–4
8. The team monitored and reassessed the situation.	Teamwork	0–4
9. The team anticipated potential actions.	Teamwork	0–4
10. The team prioritised tasks.	Task Management	0–4
11. The team followed approved standards/guidelines.	Task Management	0–4
12. Global rating.		1–10

**Table 2:** Demographic and Professional Characteristics of Participants by Group.

Category	Profession Group	CA-App Group	No-App-Group	*p*-Value
Gender (female/male)	Physicians	8/23	10/21	*p* = 0.780
	Nursing Staff	21/10	20/11	*p* = 1.000
Age (years)	Physicians	34.5 ± 6.75	37.0 ± 7.5	*p* = 0.278
	Nursing Staff	30.5 ± 12.75	32.0 ± 14.0	*p* = 0.239
Experience (years)	Physicians	5.5 ± 5.0	8.0 ± 6.0	*p* = 0.208
	Nursing Staff	8.0 ± 6.4	9.0 ± 17.9	*p* = 0.519

## Data Availability

The datasets used and analysed during the current study are available from the corresponding author on reasonable request.

## Supplementary Materials

The following supporting information can be downloaded at: https://www.mdpi.com/article/10.3390/clinpract16040069/s1, Supplement File S1: CONSORT Checklist [28].

## Abbreviations

The following abbreviations are used in this manuscript:

ACLS	Advanced Cardiac Life Support
BLS	Basic Life Support
CA	Cognitive Aid
CA-App	Cognitive Aid Application
CI	Confidence Interval
CPR	Cardiopulmonary Resuscitation
ERC	European Resuscitation Council
ICC	Intraclass Correlation Coefficient
NTS	Non-Technical Skills
RCT	Randomised Controlled Trial
ROSC	Return of Spontaneous Circulation
TEAM™	Team Emergency Assessment Measure
